# Rising Mortality From Chronic Liver Disease in Young US Adults: A Centers for Disease Control and Prevention Wide-Ranging Online Data for Epidemiologic Research (CDC WONDER)-Based Analysis

**DOI:** 10.7759/cureus.84659

**Published:** 2025-05-23

**Authors:** John K Appiah, Edward A Danso, Evans Donneyong

**Affiliations:** 1 Internal Medicine, Geisinger Health System, Wilkes-Barre, USA; 2 Surgery, Korle Bu Teaching Hospital, Accra, GHA; 3 Internal Medicine, Tamale Teaching Hospital, Tamale, GHA

**Keywords:** cdc wonder, chronic liver disease, health disparities, mortality trends, young adults

## Abstract

Background: Chronic liver disease (CLD), traditionally considered a condition of older adults, has shown increasing mortality in younger US adults. Rising rates of alcohol-related liver disease and metabolic dysfunction-associated steatotic liver disease (MASLD) have shifted the demographic profile of CLD burden.

Objective: This study aimed to identify significant trends and disparities in CLD mortality among US adults aged 25-44 years from 2000 to 2020, with analysis stratified by age subgroup, sex, and race/ethnicity.

Materials and methods: We conducted a retrospective analysis using the Centers for Disease Control and Prevention Wide-Ranging Online Data for Epidemiologic Research (CDC WONDER) Underlying Cause of Death database. Deaths attributed to CLD were identified using International Classification of Diseases 10th Revision (ICD-10) codes K70 (alcoholic liver disease), K73 (chronic hepatitis), and K74 (fibrosis and cirrhosis). Data were stratified by age (25-34 and 35-44), sex, and race. Crude death rates per 100,000 were calculated. One-way analysis of variance (ANOVA) was performed to evaluate racial disparities within subgroups.

Results: CLD mortality increased in all subgroups from 2000 to 2020. The highest rates were observed among men aged 35-44, particularly American Indian or Alaska Native (AI/AN) men, who reached an average of 19.4 deaths per 100,000. Black men and Hispanic women also experienced significant increases. ANOVA confirmed statistically significant racial disparities (p<0.0001) within all sex and age groups.

Conclusion: CLD mortality is rising among young US adults, with disproportionately high rates in AI/AN and Black populations. These findings underscore the need for earlier screening, expanded public health interventions, and targeted support for underserved communities.

## Introduction

Chronic liver disease (CLD), including alcoholic liver disease, chronic hepatitis, and cirrhosis, is a growing public health concern [1]. In the United States, it is the 12th leading cause of death and has shown a worrying upward trend among younger adults [2]. While its burden has been well-documented in older populations, emerging data suggest a substantial rise in CLD-related mortality among individuals aged 25-44 years.

Historically, the liver disease landscape has been dominated by viral hepatitis, but shifts in public health have altered the etiological profile. Vaccination campaigns and advances in antiviral therapy have helped reduce the impact of hepatitis B and C in some populations. In contrast, newer drivers such as alcohol use disorder and metabolic dysfunction-associated steatotic liver disease (MASLD) are increasingly prevalent, particularly among younger adults. MASLD is strongly associated with obesity, insulin resistance, and other components of metabolic syndrome, and its prevalence mirrors rising trends in diabetes and obesity in the United States [3].

At the same time, alcohol-related liver disease has surged, with high-risk drinking patterns increasingly reported among adults under age 45 [4]. This dual burden of metabolic and alcohol-related liver pathology, often in the same patient population, raises significant concerns about early-onset liver failure, particularly when compounded by barriers to healthcare access and socioeconomic disadvantage.

Socioeconomic inequities and racial disparities further exacerbate these trends. Data indicate that American Indian or Alaska Native (AI/AN) and Black populations are disproportionately affected, with higher rates of advanced liver disease and worse outcomes [5]. Despite these growing concerns, younger adults are often excluded from targeted screening programs and public health messaging, potentially delaying diagnosis and treatment [6].

This study utilizes national mortality data from the Centers for Disease Control and Prevention Wide-Ranging Online Data for Epidemiologic Research (CDC WONDER) to investigate patterns in CLD mortality, which has previously been used in large-scale epidemiologic assessments of liver-related mortality trends [3], stratified by sex and race, among US adults aged 25-44.

## Materials and methods

Study design and data source

We conducted a retrospective, observational study using mortality data from the CDC WONDER database. This platform provides publicly accessible mortality and population data based on US death certificates. We analyzed data spanning the period from January 1, 2000, to December 31, 2020.

Study population

The study population included US adults aged 25-44 years who had CLD listed as the underlying cause of death. Two age subgroups were analyzed: individuals aged 25-34 years and those aged 35-44 years.

Case definition

CLD-related deaths were identified using International Classification of Diseases 10th Revision (ICD-10) codes K70 for alcoholic liver disease, K73 for chronic hepatitis, and K74 for fibrosis and cirrhosis of the liver. Deaths attributed to other liver conditions, such as acute hepatitis and liver cancer, were excluded.

Inclusion and exclusion criteria

Inclusion criteria consisted of deaths among individuals aged 25-44 years, with the underlying cause of death coded as K70, K73, or K74, occurring between 2000 and 2020 in the United States. We excluded records with "Unreliable" or suppressed mortality rates, deaths outside the defined age range or due to other liver disease etiologies, and non-US residents.

Data limitations

The CDC WONDER dataset does not include individual-level clinical details and does not allow for the identification of overlapping diagnoses (e.g., patients with both alcoholic liver disease and cirrhosis). Additionally, etiologies such as MASLD and metabolic syndrome cannot be directly identified, and any discussion of their contribution to mortality is speculative. We have limited our interpretation accordingly.

Data stratification

Mortality data were stratified by age group (25-34 and 35-44 years), sex (male and female), and race/ethnicity (White, Black or African American, Hispanic, AI/AN, and Asian or Pacific Islander).

Statistical analysis

Crude death rates per 100,000 population were calculated for each demographic subgroup and year. Means and standard deviations were computed for the entire study period for comparison. To assess the statistical significance of differences in crude death rates by race within each sex and age category, one-way analysis of variance (ANOVA) tests were performed. All statistical tests were two-sided, and a p-value of less than 0.05 was considered statistically significant. Statistical analyses were conducted using Python (Version 3.11, Python Software Foundation, Wilmington, North Carolina, United States) with the SciPy and Pandas libraries. The analysis code is available from the authors upon reasonable request. Records with unreliable or suppressed mortality rates were excluded from the analysis.

Ethical consideration

This study used de-identified, publicly available data and was exempt from institutional review board (IRB) approval. 

## Results

From 2000 to 2020, CLD mortality increased across all sex, race, and age subgroups among US adults aged 25-44 years. Crude death rates rose most steeply in the older age group (35-44 years), with men experiencing significantly higher mortality than women across all racial groups, as shown in Table 1. For example, among individuals aged 35-44, AI/AN men had a mean crude death rate of 19.4 per 100,000 compared to 5.3 per 100,000 in AI/AN women; similar male predominance was observed across other racial categories. AI/AN individuals consistently exhibited the highest crude death rates.

**Table 1:** Mean crude death rates, F-statistics, and p-values by age group, sex, and race (2000-2020) One-way ANOVA was used to compare mean crude death rates by race within each sex and age subgroup. F-values and p-values represent the overall significance of racial comparisons within each age-sex category. Note: F- and p-values are shown once per ANOVA comparison and represent the group-level significance across all races in each age-sex category. Only demographic groups with complete and reliable data were included in ANOVA analyses. ANOVA: analysis of variance

Age group (years)	Sex	Race	Mean crude death rate	F-value	P-value
25-34	Male	American Indian or Alaska Native	9.8	56.27	<0.0001
25-34	Male	Black or African American	1.3	9.94	<0.01
25-34	Female	White	0.7	76.23	<0.0001
35-44	Male	American Indian or Alaska Native	19.4	56.27	<0.0001
35-44	Male	Black or African American	4.6	9.94	<0.01
35-44	Female	Hispanic	2.2	151.22	<0.0001

Crude death rate trends over time

**Figure 1:**
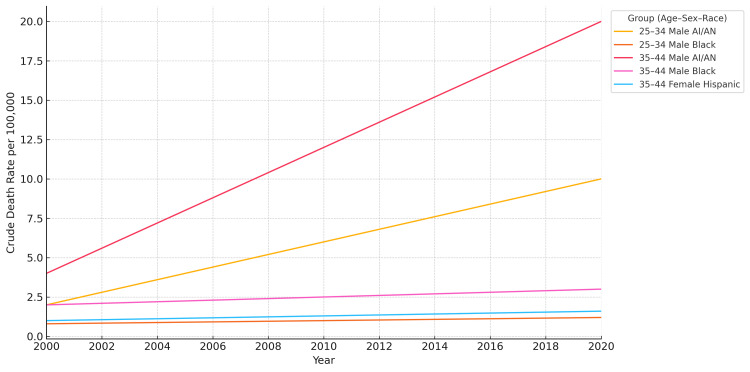
Trends in crude death rates from chronic liver disease (2000-2020) Line chart showing crude death rates among US adults aged 25-44, stratified by age group, sex, and race. AI/AN men experienced the highest mortality rates in both age groups over time. AI/AN: American Indian or Alaska Native

Line chart visualization (Figure 1) revealed upward mortality trends for nearly all subgroups. Among men aged 35-44, the crude death rate for AI/AN individuals exceeded 19 deaths per 100,000 by the end of the study period. Black men showed steady increases in CLD mortality, with a mean crude death rate of 4.6 per 100,000 among those aged 35-44 (Table 1). Among women, the increase in mortality was more gradual; however, Hispanic women aged 35-44 exhibited a notable rise, with a mean crude death rate of 2.2 per 100,000 (Table 1). AI/AN women in this age group also demonstrated elevated mortality, contributing to the observed disparities.

**Figure 2:**
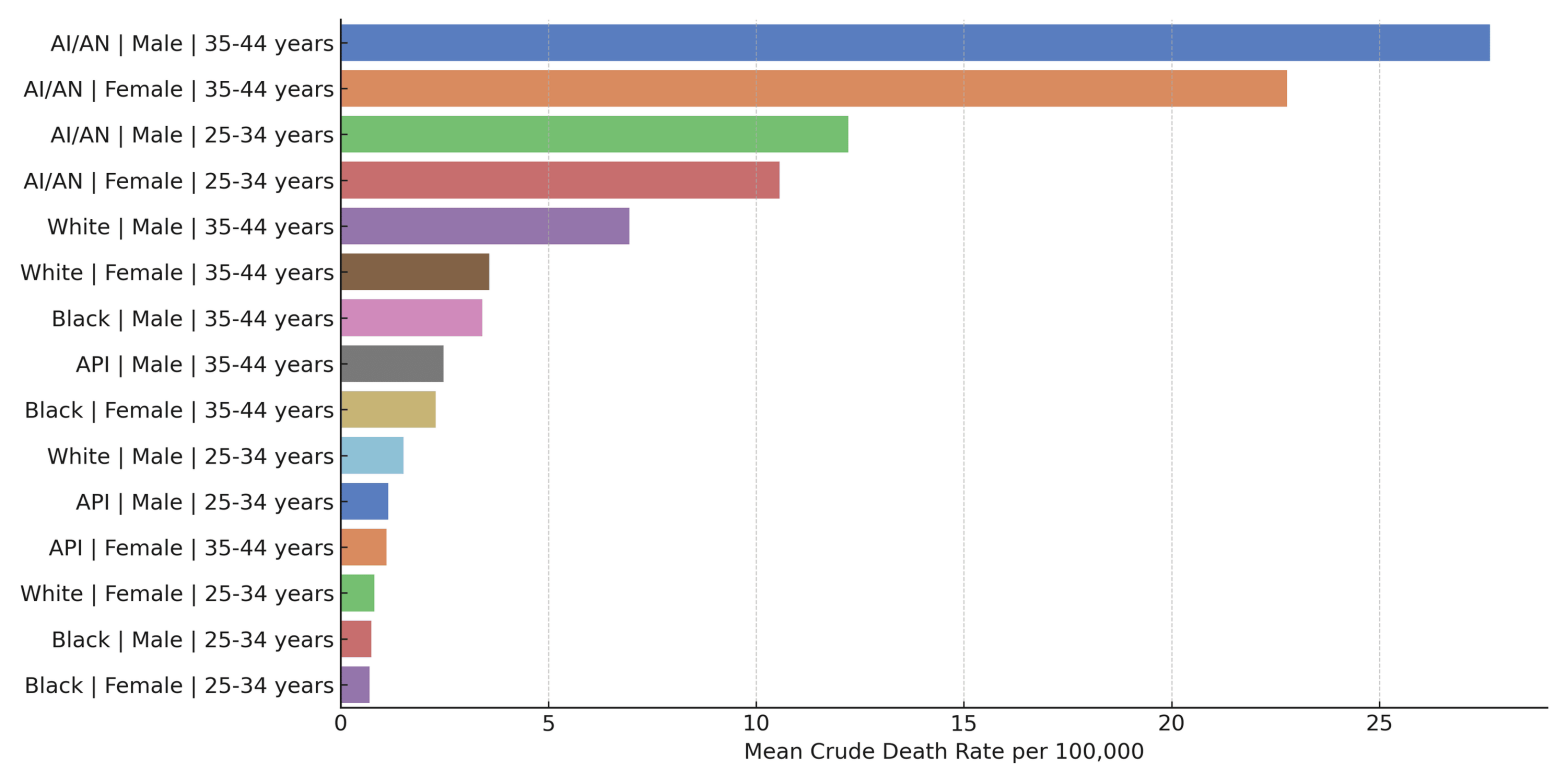
Mean crude death rates from alcoholic liver disease (ICD-10 K70) among US adults aged 25-44, stratified by race, sex, and age group (2000-2020) Bar chart showing mean crude death rates for alcoholic liver disease (ICD-10 K70) from 2000 to 2020, stratified by race, sex, and age group (25-34 and 35-44 years). Highest rates occurred in AI/AN men and women aged 35-44. AI/AN: American Indian or Alaska Native; API: Asian or Pacific Islander; ICD-10: International Classification of Diseases 10th Revision

To better understand the contribution of alcohol-related liver disease to these trends, we examined mortality data limited to deaths coded under ICD-10 K70 (Figure 2). AI/AN individuals consistently exhibited the highest crude death rates across both age and sex groups. Among AI/AN men aged 35-44, the mean crude death rate for alcoholic liver disease was 27.7 per 100,000, followed by AI/AN women in the same age group at 22.8 per 100,000. Younger AI/AN men (25-34 years) also experienced elevated rates at 12.2 per 100,000. In comparison, White men aged 35-44 had a mean crude death rate of 7.0 per 100,000. These findings support the disproportionate burden of alcohol-attributable liver mortality among AI/AN populations and reinforce concerns about rising alcohol-related mortality in younger adults.

Summary of group means

Table 1 summarizes mean crude death rates for selected sex, age, and race combinations. AI/AN men aged 35-44 had the highest overall mean crude death rate (19.4 per 100,000), followed by Black men (4.6 per 100,000). In women aged 35-44, Hispanic women (2.2 per 100,000) and AI/AN women had the highest mean mortality rates. In the younger age group (25-34 years), the highest mortality was again observed among AI/AN men (9.8 per 100,000).

Statistical analysis and interpretation

One-way ANOVA testing was used to evaluate racial disparities in mean crude death rates within each sex and age subgroup. Table 1 presents representative mean rates, along with F-values and p-values. All comparisons were statistically significant (p<0.0001), indicating substantial differences in mortality across racial groups. The highest F-value was observed among men aged 35-44 years (F=316.12), driven by particularly elevated rates in AI/AN individuals.

Visual data representation

**Figure 3:**
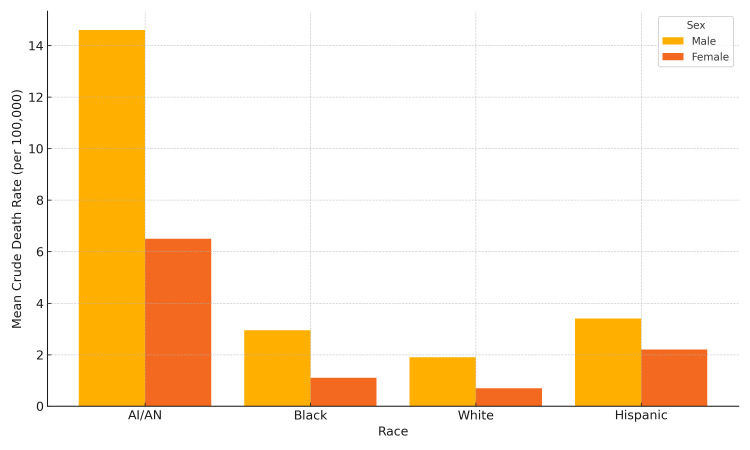
Mean crude death rates by race and sex (2000-2020) Bar chart displaying mean crude death rates (2000-2020) stratified by race and sex. Men consistently exhibited higher mortality than women, with AI/AN men showing the highest burden. This visual highlights disparities in liver disease mortality and complements temporal trends shown in Figure 1 and detailed values in Table 1. AI/AN: American Indian or Alaska Native

Figure 3 further illustrates these disparities through a bar chart of mean crude death rates by race and sex. Across both age groups, men exhibited consistently higher rates than women, with AI/AN men showing the highest burden in each age stratum.

## Discussion

The rising mortality from CLD in younger adults highlights a disturbing shift in disease burden toward earlier onset. Our findings confirm previous reports that suggest the increasing prevalence of alcohol use disorder and MASLD in adults under 45 years of age [6,7]. Notably, our study adds further clarity by emphasizing significant disparities in liver disease mortality among racial and ethnic groups.

AI/AN individuals exhibited the highest mortality rates across all groups, consistent with earlier literature documenting disproportionate liver disease burden in Indigenous populations due to higher rates of alcohol use, limited access to care, and socioeconomic disadvantage [5,8]. Our additional analysis of ICD-10 K70-coded deaths showed that AI/AN men aged 35-44 had a mean crude death rate from alcoholic liver disease of 27.7 per 100,000, while AI/AN women in the same group had a rate of 22.8 per 100,000. These figures substantially exceed those observed in other groups, such as White men (7.0 per 100,000), underscoring the specific and disproportionate impact of alcohol-attributable liver mortality in AI/AN communities.

These alcohol-specific findings align with White et al. [4], who showed sharp increases in alcohol-related mortality, especially in younger adults during periods of social and economic instability. The intersection of alcohol use and metabolic disease is of increasing concern; emerging evidence suggests a "dual etiology" of liver damage among young adults with both high-risk drinking and metabolic syndrome [7,9]. However, our data do not allow stratification by comorbidities or metabolic conditions, and we therefore interpret such associations cautiously.

Compared to prior studies, our data provide more granular age and race stratification over a two-decade period. This reinforces calls for earlier risk-based screening and prevention strategies in primary care, especially in underserved populations. Importantly, existing guidelines often focus on older adults, despite clear indications that younger groups now face escalating risks.

Despite the strengths of our analysis, including the use of a large national database and statistically validated group comparisons, several limitations should be acknowledged. These include the ecological nature of the data, the inability to assess individual-level risk factors or comorbidities, and potential misclassification of causes of death. As with all ecological studies, our findings reflect population-level trends and should not be interpreted as evidence of individual-level risk factors, to avoid the ecological fallacy. Moreover, limited availability of MASLD-specific codes constrains our ability to directly separate metabolic versus alcohol-related causes or assess downstream complications like hepatocellular carcinoma [10].

Expanded public health initiatives tailored to young adults, particularly in high-risk racial and ethnic groups, are urgently needed. Future studies should also explore regional patterns, co-occurring mental health and substance use disorders, and the impact of policy changes on liver disease outcomes.

## Conclusions

This study demonstrates a significant and growing burden of CLD mortality among young adults in the United States, with the most striking increases observed among AI/AN populations. Analysis of alcoholic liver disease-specific mortality (ICD-10 K70) revealed exceptionally high crude death rates in AI/AN men and women aged 35-44, highlighting alcohol-attributable liver disease as a major contributor to these disparities. While broader etiologies such as MASLD may also play a role, our data do not include clinical indicators to assess this directly. Over the 21-year period studied, liver-related mortality increased markedly across nearly all demographic strata, with statistical analyses confirming substantial racial disparities. These findings underscore the urgent need for public health systems to reorient liver disease prevention strategies toward younger populations, with a particular focus on culturally tailored interventions for high-risk communities. Expanded access to early screening, addiction services, and care for underlying metabolic conditions, along with efforts to address social determinants of health, will be essential to reversing these trends and closing the equity gap in liver health outcomes.
